# Comparing Dermatoscopic Features With Slit Skin Smear and Histopathology in Diagnosis of Cutaneous Leishmaniasis

**DOI:** 10.7759/cureus.35336

**Published:** 2023-02-22

**Authors:** Shafia Memon, Najia Ahmed, Syed Arbab Shah, Arfan ul Bari, Muhammad Rahim, Omer Farooq, Mohammad Nasir Memon

**Affiliations:** 1 Department of Dermatology, Bahria University Medical and Dental College, Pakistan Navy Station (PNS) Shifa Hospital, Karachi, PAK; 2 Department of Dermatology, Bahria University of Health Sciences, Pakistan Navy Station (PNS) Shifa Hospital, Karachi, PAK; 3 Department of Dermatology, Combined Military Hospital and Army Medical College, Rawalpindi, Islamabad, PAK; 4 Dermatology, Pakistan Navy Station (PNS) Shifa Hospital, Karachi, PAK; 5 Ophthalmology, Bahria University of Health Sciences, Pakistan Navy Station (PNS) Shifa Hospital, Karachi, PAK; 6 Biostatistics, Get Solutions 360, Karachi, PAK

**Keywords:** star-burst pattern, yellow tears, histopathology, slit smear, cutaneous leishmaniasis, dermatoscopy

## Abstract

Objective: To evaluate dermatoscopic features of cutaneous leishmaniasis and to compare its diagnostic accuracy against slit skin smear and skin histopathology.

Methods: This cross-sectional study was conducted at the Department of Dermatology, Pakistan Navy Station (PNS) Shifa Hospital, Karachi, Pakistan, from August 2021 to August 2022. A total of 200 lesions from 70 patients of cutaneous leishmaniasis diagnosed with slit skin smear for Leishmania-Donovan (LD) bodies and skin biopsy were included via non-probability consecutive sampling technique. Dermatoscopic evaluation was performed via a handheld dermatoscope (DELTA 20T; HEINE, Gilching, Germany) on 10x magnification. All dermatoscopic images were analyzed by two different observers who had command of dermatoscopy. Data analysis was done using Statistical Package for the Social Sciences SPSS version 27 (IBM Corp., Armonk, NY, USA).

Results: Common dermatoscopic findings were erythema 200 (100%), hyperkeratosis 140 (70%), crusting 50 (25%), ulceration 42 (21%), milia-like structure 58 (29%), tear drop-like structure 46 (23%), yellow tears 70 (35%), and white starburst pattern 68 (34%). Less common findings were yellow hue 28 (14%), orange areas 26 (13%) and scar seven (3.5%). Vascular structures frequently observed were linear vessels 109 (54.5%), dotted vessels 80 (40%), and hairpin vessels 61 (30.5%); less common findings were comma-shaped vessels 52 (26%), arborizing vessels 20 (10%), crown vessels nine (4.5%). Comparison of dermatoscopic features was done with slit skin smear for LD bodies (p value = 0.003 ) and histopathology (p value = 0.001).

Conclusions: Dermatoscopy is a non-invasive technique that is helpful in diagnosing cutaneous leishmaniasis, saving time in making rapid diagnosis and saving the need to undergo extensive invasive investigation. Yield of dermatoscopy was comparable to slit smear for LD bodies and histopathology and was found to be effective in making rapid diagnosis with significant accuracy (p value <0.05).

## Introduction

Leishmaniasis is an infectious disease caused by flagellate protozoa of the genus Leishmania that are transmitted by vector sandflies of the genera Phlebotomus (old world) and Lutzomyia (new world) [1]. More than 12 million people are affected by the disease globally with cutaneous leishmaniasis (CL) being the most common form [2]. About 21,000-35,000 cases of both zoonotic CL (ZCL) and anthroponotic CL (ACL) are reported in Pakistan [3]. Various tests have been used for diagnosing CL, but parasitological diagnosis remains the gold standard in diagnosing leishmaniasis, typically done via Giemsa-stained smears from lesional skin, immunology, culture, histopathology, molecular techniques like polymerase chain reaction (PCR) and DNA probing [4-6]. Dermatoscopy, also known as ‘epiluminoscopy’, is a non-invasive diagnostic technique that facilitates visualization of the epidermis and superficial dermis [7]. It has been mainly used for evaluation of pigmented skin lesions like melanoma [7-9], but now it has taken its place as a diagnostic tool in almost all fields of dermatology, i.e. skin cancers like squamous cell carcinoma, basal cell carcinoma, infections and inflammatory dermatoses [10-14]. Dermatoscopy requires a powerful lighting system and high-quality magnifying lens with convenient attachments which allow video or still photography, which can be used to assess treatment response of the patient on follow-up visits [7].

Our study aimed to compare dermatoscopic findings of CL with slit smear for Leishmania-Donovan (LD) bodies and skin biopsy as the dermatoscope is non-invasive, consumes less time, gives immediate results and in experienced hands the yield is excellent. It can also be of great value, where skin biopsy can be difficult to perform due to lack of facility or chances of scarring after the procedure. This study is unique as no such study has been conducted before that compares the yield of dermatoscopic features of CL with slit skin smear and skin biopsy.

## Materials and methods

This cross-sectional analytical study was conducted at the Dermatology Department at Pakistan Navy Station (PNS) Shifa Hospital, Karachi, Pakistan, over a period of one year from August 2021 to August 2022, after approval from the institutional ethical committee (ERC/2021l/DERMA/44). The sample size was calculated using the OPENEPI calculator using a prevalence of 90.6%, margin of error=5%, confidence level=95% was calculated 131 lesions. A total of 200 lesions from 70 patients of cutaneous Leishmaniasis, diagnosed with slit skin smear for LD bodies and skin biopsy were included via non-probability consecutive sampling technique. Written informed consent was taken from each patient after explaining the study.

Inclusion criteria

Patients aged 18 to 50 years who had leishmania lesions (papules, nodules, plaques or noduloulcertive plaques) for more than six months, irrespective of gender, and who did not receive topical, intralesional or systemic anti-leishmania treatment in the past two months were included in the study.

Exclusion criteria

Patients who had lesions (papules, nodules, plaques or noduloulcertive plaques) for more than six months and received any topical, intralesional or systemic anti-leishmania treatment in the past two months or more were excluded from this study.

All the included lesions were individually examined and assessed. Dermatoscopy was done using a DELTA 20T (HEINE, Gilching, Germany) with a ×10 magnification and all photographs were taken by iPhone 13 pro. All relevant information was taken. At least three dermatoscopic images were taken from each lesion. All images were analyzed by two individual observers to avoid any kind of bias. For better quality images, each lesion was wiped using 70% isopropyl alcohol and it was also used when using dermatoscope between different patients to avoid contamination. Gentle pressure was applied using the dermatoscope to protect vascular structures and prevent vascular collapse.

Dermatoscopic characteristics of the lesions, like erythema, crusting, hyperkeratosis, ulceration, teardrop‑like structures, milia‑like cyst, yellow tears, yellow hue white‑starburst pattern, scar, and orange areas were evaluated. Vascular structures in the lesions were evaluated based on morphology including dotted vessels, linear vessels, hairpin‑like vessels, arborizing vessels, comma-shaped vessels and crown vessels.

Statistical evaluation were performed using the Statistical Package for the Social Sciences (SPSS) version 27 (IBM Corp., Armonk, NY, USA). Frequencies and percentages were utilized for categorical data and Mean + SD was used for descriptive statistics. A one-way ANOVA was used to compare the statistical difference among dermatoscopy of CL, slit skin smear and skin biopsy. P<0.05 was considered statistically significant.

## Results

Clinical and demographic characteristics of the 70 patients are elaborated inTable 1. The most prevalent dermatoscopic findings were erythema 200 (100%), hyperkeratosis 140 (70%), crusting 50 (25%), ulceration 42 (21%), milia-like structure 58 (29%), tear drop-like structure 46 (23%), yellow tears 70 (35%), and white starburst pattern 68 (34%). Less common findings were yellow hue 28 (14%), orange areas 26 (13%) and scar 7 (3.5%). Vascular structures frequently observed were linear vessels 10 (54.5%), dotted vessels 80 (40%), and hairpin vessels 61 (30.5%), and less common findings were comma-shaped vessels 52 (26%), arborizing vessels 20 (10%), and crown vessels 1 (0.5%).

**Table 1:** Clinical and Demographic Characteristics

Characteristics	(n=200), n% /mean
Gender and Age	
Female	12 (17)
Male	58 (82)
Age (in years) (mean ± SD)	24.05 ± 18.9
Fitzpatrick skin type	
III	11 (15.7)
IV	41 (58.6)
V	18 (25.7)
No. of Lesions	
1	41(20)
2	40 (19.8)
3	52 (26)
4	30 (15)
5	37 (18.5)
Type of Lesions	
Papular	59 (29.5)
Nodular	40 (20)
Nodulo-ulcerative	52 (26)
Plaque	49 (24.5)
Location of Lesions	
Face (n= 82)	
Forehead	33 (16.5)
Periorbital Region	8 (4)
Ear	7 (3.5)
Nose	14 (7)
Lip	15 (7.5)
Chin	5 (2.5)
Neck (n=33)	
Anterior side of neck	19 (9.5)
Posterior side of neck	3 (1.5)
Lateral side of neck	11(5.5)
Upper extremities (n=41)	
Arm	4 (2)
Forearm	11 (5.5)
Hand	26 (13)
Lower extremities (n=44)	
Thigh	21 (10.5)
Leg	8 (4)
Foot	15 (7.5)

Dermatoscopic features when assessed according to region of body involved includes tear drop structures 77 (38.5%) and dotted 28 (14%), comma-shaped vessels 24 (12%), and arborizing vessels 15 (7.5%), commonly found on face; milia-like structures 15 (7.5%), white star burst pattern 15 (7.5%), and comma-shaped vessels 30 (15%) were most frequent findings in neck; tear drop structure 54 (27%), white star burst pattern 33 (16.5%) and arborizing vessels 33 (16.5%) on upper extremities; milia-like structures and tear drop structures 34 (17%) and comma-shaped vessels and crown vessels 28 (14%) on lower extremities.

Dermatoscopic lesions when evaluated according to the type of lesion, erythema was found to be present in all lesions, milia-like structures, starburst pattern and linear vessels were more common in papular type. Crusting, milia-like structures and hair pin vessels were common in plaque type; yellow tear, milia-like structures, starburst pattern and linear vessels in nodule and hyperkeratosis, crusting, ulceration with hair pin and linear vessels in nodulo-ulcerative type.

Dermatoscopic correlation was done with slit smear for LD bodies with p value (p = 0.003) and histopathology (p = 0.001), which was found to be significant. Comparison of dermatoscopic features with histopathological diagnosis and slit smear for LD bodies is shown in Table 2.

**Table 2:** Comparison of dermatoscopic features of CL with slit smear and histopathological diagnosis (n=200) CL: cutaneous leishmaniasis

Disease	Histopathological diagnosis) (n/%)	Slit skin smear (n/%)	Dermatoscopic Diagnosis (n/%)	Positive histopathological and Dermatoscopic Correlation (n/%)	Positive correlation with slit smear and dermatoscopic features (n/%)
Leishmaniasis	69 (98.6%)	62 (88.6%)	64 (91.4%)	90%	95%

	Mean ± SD	F ratio	P value
Slit smear	25.9 ±14.3	12.8	0.003
Skin Biopsy	36.2 ± 15.6	57.7	0.001

Dermatoscopy Features	Slit Smear (Mean ± SD)	Skin Biopsy (Mean ± SD)	F ratio	P value
Erythema	11.9 ± 1.01	48.76 ± 24.4	1.497	0.000
Hyperkeratosis	3.76 ± 1.271	39.7 ± 10.7	1.036	0.003
Yellow tears	44.7 ± 1.277	30.5 ± 1.37	1.476	0.002
White starburst pattern	48.8 ± 1.231	2.65 ± 1.52	1.176	0.001
Tear drop like structures	49.0 ± 1.47	57.4 ± 1.03	1.29	0.002
Linear vessels	42.7 ± 1.67	14.5 ± 1.09	1.646	0.000
Dotted vessels	41.77 ± 1.7	18.2 ± 1.82	1.476	0.000
Arborizing vessels	43.7 ±1.27	17.6 ± 1.34	1.029	0.001

## Discussion

There are five studies done previously to evaluate the dermatoscopic patterns of CL [15-20]. Our study showed that erythema was found in 100% of lesions, which were dermatoscoped like in previous studies [15-20], with difference in background red color 50% were dull red, 23% were dusky red and 27% were bright red mostly face and neck lesions.

**Figure 1:**
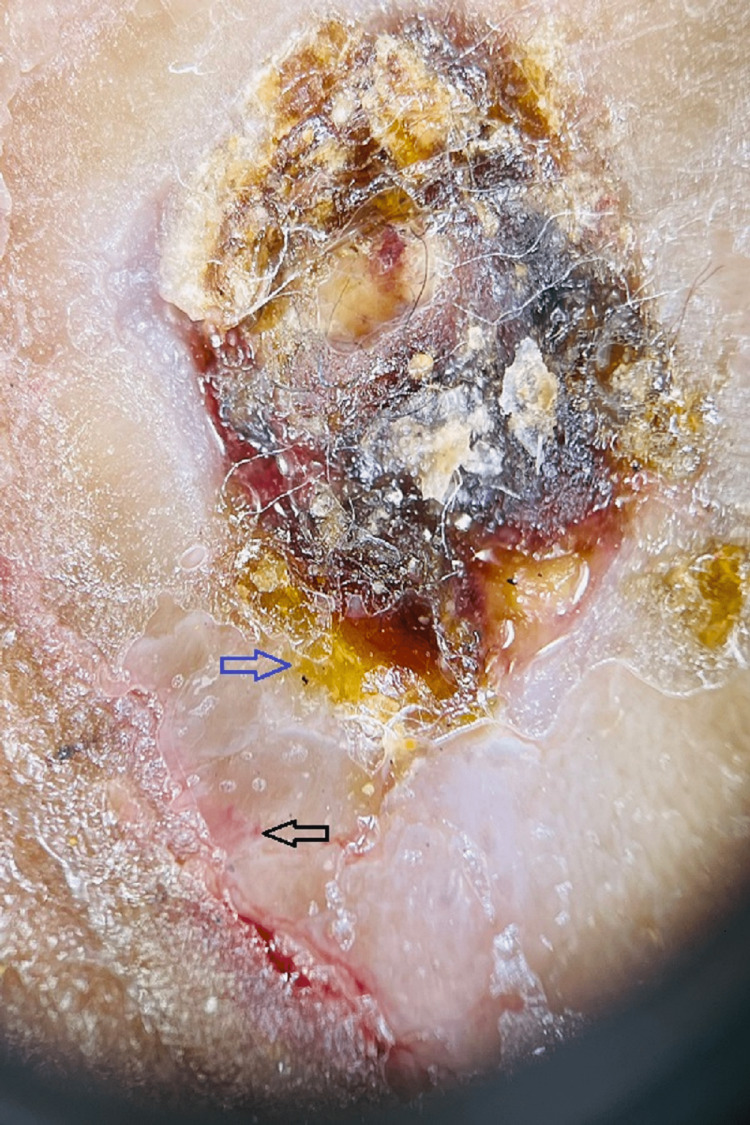
The center shows hyperkeratosis crusting and ulceration. Blue arrow shows yellow hue and black arrow shows polymorph vessels.

Hyperkeratosis was seen in 70% of lesions (Figure 1), which was seen less in other studies [15-20], because 24.5% plaque-type lesions were evaluated in our study. Yellow tears were appreciated in 35% of lesions**.**

Twenty-one percent ulceration was appreciated, which is as comparable to Yücel et al. study as 26% nodulo-ucerative lesions were dermatoscoped in our study [18]. Forty-six (23%) tear drop structures were appreciated in our study exclusively on face (p = 0.002), neck (p = 0.004), and upper extremities (p = 0.012).

**Figure 2:**
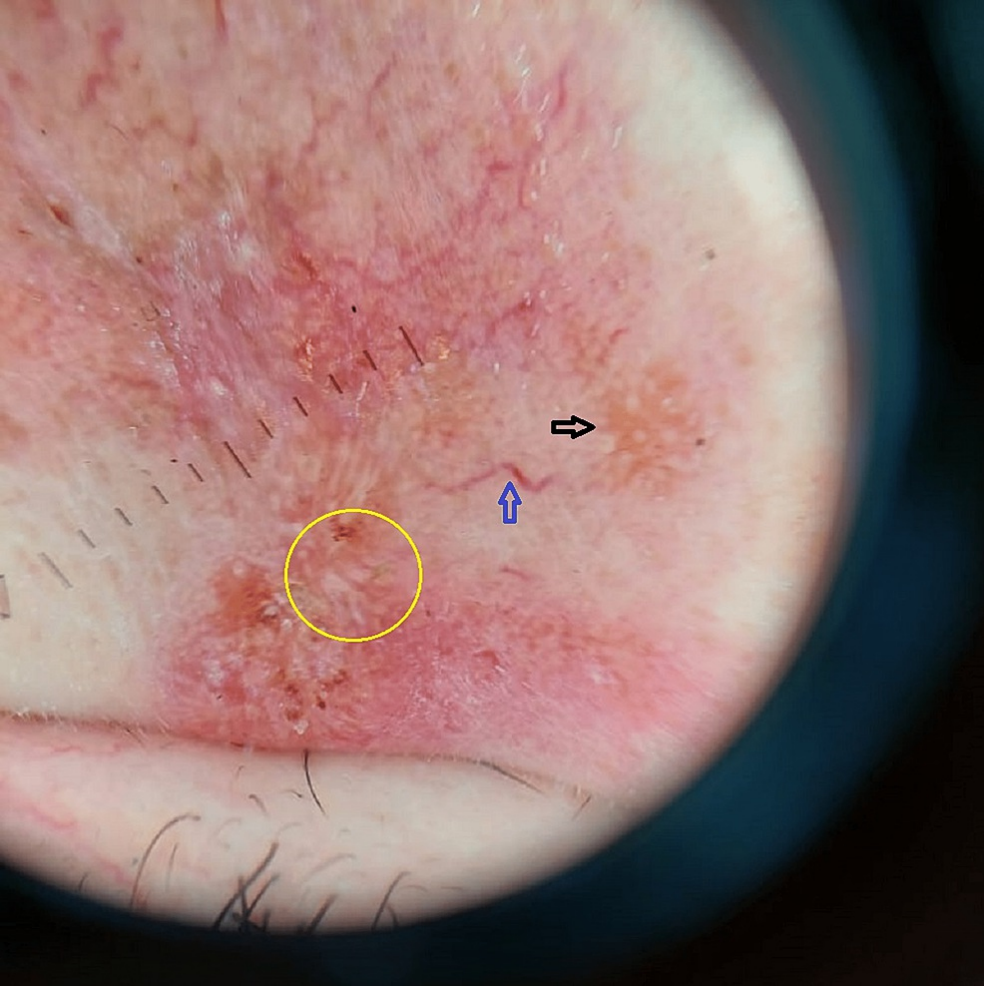
Black arrow shows milia-like structures, blue arrow shows arborizing vessel and yellow circled area demonstrates tear-drop structures.

Fifty-eight (29%) milia-like structures were found in this study, which were present more in plaque, nodular and nodulo-ulcerative lesions but not in papular-type lesions as shown in Figure 2. Sixty-eight (34%) white starburst pattern appreciated in our study more in nodular (10.5%) and nodulo-ulcerative lesions (14%). In vascular structures, linear vessels 54.5% were predominantly found vessels as in previous studies, in all type lesions more on lower extremities and upper extremities.

Other vascular structures were dotted vessels 40%, mostly in plaque (p <0.0001). Hairpin vessels 30.5%, 26% comma-shaped vessels and 10% arborizing vessels were appreciated, least common findings were crown vessels appreciated only in a single lesion which was not appreciated in any previous studies [17-21]. A comparison of our study with previous studies is shown in Table 3.

**Table 3:** Comparison of this study with similar previous studies conducted - is not reported or evaluated.

	Our Study	Yucel et al. [18]	Eroglu et al. [20]	Taheri et al. [19]	Llambrich et al. [15]
Dermatoscopic Features	(Pakistan)	(Turkey)	(Turkey)	(Iran)	(Spain)
	(n=200), n (%)	(n=145), n (%)	(n=225), n (%)	(n=144), n (%)	(n=26), n (%)
General Features					
Erythema	200 (100)	145(100)	225 (100)	118 (81.9)	26 (100)
Hyperkeratosis	140 (70)	-	120 (53.2)	48 (33.3)	13 (15.7)
Crusting	50 (25)	51 (35.2)	79 (35.1)	-	-
Ulceration	42 (21)	51 (35.2)	8 (3.5)	85 (59)	12 (46)
Milia like structure,	58 (29)	58 (40)	133 (59.1)	60 (41.7)	14 (53)
Tear drop like structures	46 (23)	-	60 (26.7)	7 (4.9)	-
Yellow tears	70 (35)	19 (13.1)	76 (17.1)	63 (43.8)	-
White starburst pattern	68(34)	27 (18.6)	76(33.8)	87 (60.4)	10 (38)
Yellow hue	28 (14)	-	25 (11.1)	-	-
Orange areas	26 (13)	-	20 (8.9)	-	-
Scar	7 (3.5)	4 (2.8)	-	-	-
Vascular structures					
Linear vessels	109 (54.5)	78 (24)	113(24)	54 (37.5)	5 (19)
Dotted vessels	80 (40)	23 (10)	89 (10)	88 (10.4)	3 (11)
Hairpin vessels	61 (30.5)	25 (30.5)	74 (30.5)	54 (30.6)	15 (57)
Coma shaped vessels	52 (26)	6 (4.1)	49(26)	43 (61.1)	14 (53)
Arborizing vessels	20 (10)	53 (5)	70 (5)	15 (29.9)	19 (73)
Crown vessels	1 (0.5)	-	-	-	-

Llambrich et al. first dermatoscoped 26 lesions of CL in 25 patients and found teardrop‑like structures in 53% [15]. Ayhan et al. detected in his study teardrop‑like structures in 42.5% of the lesions, and teardrop‑like structures were appreciated only in lesions located on the face and the posterior and lateral sides of the neck, not on body sites [17]. Yücel et al. detected teardrop‑like structures in 40% of the lesions in that study, and these structures were frequently appreciated in nodular lesions [18]. Eroglu et al.'s study detected teardrop‑like structures in 59.1% of the lesions, particularly observed in new papular lesions without crust (p = 0.728) [20]. Histopathologically, teardrop‑like structures were considered to be follicular fillings plugged with keratin formed due to pressure on the hair follicle from the sides. These follicular fillings are also appreciated in non-pigmented actinic keratosis, forming a strawberry pattern. In the strawberry pattern in non-pigmented actinic keratosis, however, these keratin plugs follicular filling are not inside the structure but around the pseudoweb with surrounding erythema without ulcer or crust in the center. Differences like this may help with the dermatoscopic differential diagnosis of CL. Parakeratotis with hyperkeratosis visualized as white starburst‑like patterns, first detected by Llambrich et al. in CL, was detected in 38% of the lesions. Taheri et al. in their study found this in 60.4% of the lesions, mainly nodular lesions on the upper extremities. Yücel et al. evaluated this finding in 19% of the lesions, frequently in nodulo-ulcerative lesions [18]. In Eroglu et al.'s study white burst pattern and yellow tears were frequently observed previously under-reported in literature [20], arborizing vessels were also not frequently found on dermatoscopy of CL, visualized more on face, upper extremities and neck.

This study is unique, because along with mentioning dermatoscopic features of CL, it also correlates dermatoscopic features of CL with slit skin smear and histopathology. Our study found significant diagnostic accuracy of dermatoscopy in comparison with slit skin smear and histopathology. The P values and mean values of ANOVA show that dermatoscopic features of CL are essentially equal to slit smear and skin biopsy for the diagnosis of cutaneous leishmaniasis. Comparing the skin biopsy, dermatoscopy and slit smear, the results are statistically significant for all three variables with all p values less than 0.05.

## Conclusions

This study concluded that dermatoscopy is a non-invasive, rapid tool in diagnosing CL. Comparison of dermatoscopic features to slit skin smear for LD bodies and histopathology was found significant with p value < 0.05. Diagnostic accuracy was found to be comparable to slit skin smear and histopathology, helpful in making rapid diagnoses without undergoing invasive procedures. No characteristic features are attributed to CL, but the constellation of features on dermatoscopy which are helpful to rule out differential diagnosis easily. Multi-center studies on larger populations should be done to evaluate the effectiveness of dermatoscopy in diagnosing cutaneous leishmaniasis more accurately.
